# Kayadiol exerted anticancer effects through p53-mediated ferroptosis in NKTCL cells

**DOI:** 10.1186/s12885-022-09825-5

**Published:** 2022-07-02

**Authors:** Cuiying He, Chengzhao Wang, Haisheng Liu, Baoen Shan

**Affiliations:** 1 Department of Hematology, The Fourth Hospital of Hebei Medical University, NO.169, TianShan Street, Shijiazhuang, Hebei 050035 People’s Republic of China; 2 Research Center and Tumor Research Institute, The Fourth Hospital of Hebei Medical University, NO.12, Jiankang Road, Shijiazhuang, Hebei 050011 People’s Republic of China; 3 Hebei Medical University, Shijiazhuang, China; 4 College of Basic Medicine, Hebei Medical University, Shijiazhuang, China

**Keywords:** Kayadiol, Ferroptosis, p53, NKTCL

## Abstract

**Background:**

Extranodal natural killer/T cell lymphoma (NKTCL) is a highly aggressive type of non-Hodgkin lymphoma that facing the treatment challenges. Natural compounds are important sources for drug development because of their diverse biological and chemical properties, among which terpenoids have strong anticancer activities.

**Methods:**

The human NK/T cell lymphoma cell line YT and peripheral blood lymphocytes isolated from NKTCL patients were treated with different concentrations of kayadiol. Then, the following experiments were performed: CCK-8 assay for cell viability, reactive oxygen species (ROS) and glutathione (GSH) assay and co-treatment with NAC, reduced GSH, or ferrostatin-1 for ferroptosis, the proteome profiling for elucidating signaling pathways, and western blot for the expression of p53, SCL7A11, and GPX4. siRNA and CRISPR/Cas9 plasmid for p53 knockout was designed and transfected into YT cells to evaluate the causal role of p53 in kayadiol-induced ferroptosis. The synergistic effect was evaluated by CCK8 assay after co-treatment of kayadiol with L-asparaginase or cisplatin.

**Results:**

In this study, we found that kayadiol, a diterpenoid extracted from *Torreya nucifera*, exerted significant killing effect on NKTCL cells without killing the healthy lymphocytes. Subsequently, we observed that kayadiol treatment triggered significant ferroptosis events, including ROS accumulation and GSH depletion. ROS scavenger NAC, GSH, and ferroptosis inhibitor ferrostatin-1 (Fer-1) reversed kayadiol-induced cell death in NKTCL cells. Furthermore, kayadiol decreased the expression of SLC7A11 and GPX4, the negative regulatory proteins for ferroptosis. We then demonstrated that p53 was the key mediator of kayadiol-induced ferroptosis by SLC7A11/GPX4 axis through p53 knockout experiments. In addition, kayadiol exerted a synergistic effect with L-asparaginase and cisplatin in NKTCL cells.

**Conclusion:**

Taken together, our results suggested that the natural product kayadiol exerted anticancer effects through p53-mediated ferroptosis in NK/T cell lymphoma cells. Hence, it can serve as an effective alternative in the treatment of NK/T cell lymphoma, especially for patients exhibiting chemoresistance.

**Supplementary Information:**

The online version contains supplementary material available at 10.1186/s12885-022-09825-5.

## Introduction

Extranodal natural killer /T-cell lymphoma (NKTCL) is a subtype of non-Hodgkin lymphoma derived from mature T cells or natural killer (NK) cells. NKTCL is a highly aggressive neoplasm with a poor prognosis. Currently, treatment for NKTCL mainly relies on chemotherapy and radiotherapy, and L-asparaginase (L-Asp)-based treatment regimen is considered as the first-line option. Although most patients with NKTCL can achieve clinical remission after L-Asp-containing regimens and radiotherapy, the long-term overall survival (OS) remains poor, especially for patients with advanced stage and refractory/relapsed diseases [1, 2]. Despite the recent progress in developing new molecularly targeted drugs and antibodies, therapeutic outcomes remain unsatisfactory. Therefore, identifying new anticancer drugs is critical to improve the prognosis of NKTCL patients.

Natural compounds are important sources for drug development because of their diverse biological and chemical characteristics. Many drugs derived from natural compounds have been clinically used, among which terpenoids have strong anticancer activities [3, 4]. By screening some rarely reported natural terpenoids, we found that kayadiol had a significant killing effect on NKTCL cells. Kayadiol is a natural diterpenoid extracted from the pulp of *Torreya nucifera*. Little of the property of kayadiol has been studied, and its anti-cancer effect has only been reported in some cell lines [5]. The antitumor effects and the mechanism of kayadiol in NK/T cell lymphoma remain unknown.

Ferroptosis is a newly discovered form of programmed cell death, which characterized by accumulation of lipid peroxides-reactive oxygen species (ROS) within the cell [6, 7]. Ferroptosis is usually caused by iron-dependent oxidative damage, and the classical ferroptosis pathway is usually induced by the failure of membrane protective mechanisms against peroxidative damage. Glutathione peroxidase (GPX4) uses glutathione (GSH) as a reductant to catalyze the reduction of lipid hydroperoxides to non-toxic lipid alcohols, thereby protecting cells from lipid peroxidative damage. GSH depletion or GPX4 inactivation leads to the accumulation of ROS, which induces classical ferroptosis [8, 9]. Increasing evidence suggests that ferroptosis plays an important role in various human diseases, including tumorigenesis [10, 11]. Activation of ferroptosis in tumors would be a potential treatment strategy.

In this study, we found that kayadiol exerted a significant killing effect on NKTCL cells without killing healthy lymphocytes. Next, we demonstrated for the first time that ferroptosis was consequent to kayadiol-induced cell death, and p53 was a critical mediator of kayadiol-induced ferroptosis. Furthermore, kayadiol enhanced the sensitivity of NKTCL cells to L-Asp and cisplatin. Taken together, our results suggested that the natural product kayadiol exerted anticancer effects through p53-mediated ferroptosis in NKTCL cells and could be an effective alternative for NK/T cell lymphoma therapy, especially for patients exhibiting chemoresistance.

## Methods

### Cells and chemicals

The human NK/T cell lymphoma cell line YT was kindly provided by Dr. Mingzhi Zhang, Zhengzhou University First Affiliated Hospital, Zhengzhou University, Zhengzhou, Henan, China. Cells were maintained in Roswell Park Memorial Institute 1640 (RPMI1640; Gibco, Tulsa, OK, USA) medium, supplemented with 10% fetal bovine serum. Kayadiol was kindly provided by Dr. Mei Dong, Hebei Medical University, Shijiazhuang, Hebei, China. The following chemicals were obtained from commercial sources: reduced L-glutathione (GSH, HY-D0187; MCE LLC., Monmouth Junction, NJ, USA), N-acetylcysteine (NAC, HY-B0215; MCE LLC.), ferrostatin-1(Fer-1, HY-100579; MCE LLC.), cisplatin (HY-17394; MCE LLC.), and L-Asp (HY-P1923, MCE LLC.).

### Peripheral blood lymphocytes (PBLs) isolation

Peripheral blood lymphocytes (PBLs) were isolated from NKTCL patients and healthy donors using a Human Peripheral Blood Lymphocyte Separation Solution (Tbdscience, China). All participants provided their written informed consent and the procedure was accordance with the Ethic Committee of the Forth Hospital of Hebei Medical University.

### Cell viability assay

Cell viability, expressed as cell proliferation, was measured using a cell counting kit-8 (CCK-8) assay. YT cells or PBLs were added to 96-well microtiter culture plates and stimulated with different concentrations of kayadiol. At the end of each cell culture period, cells were incubated with the CCK-8 solution for an additional 2 h, and the absorbance was detected at 450 nm by a Multiskan Sky Microplate Spectrophotometer (Thermo Fisher Scientific, Eugene, OR, USA).

### ROS assay

The ROS levels were detected using a ROS assay kit (Wanleibio, Shenyang, China). After stimulation with kayadiol, YT cells were incubated with DCFH-DA fluorogenic probe for 30 min and fluorescence intensity was measured using a fluorescence microplate reader (Thermo Fisher Scientific, Eugene, OR, USA).

### GSH assay

The GSH levels were detected using a GSH and GSSG assay kit (Beyotime, Haimen, Chian) and normalized on the basis of cell numbers, according to the manufacturer’s instructions.

### Co-treatment of kayadiol and ferroptosis inhibitors

YT cells were added to 96-well microtiter culture plates and stimulated with kayadiol (12.5 μM) with or without ROS scavenger NAC (5 mM), reduced GSH (1 mM), or ferroptosis inhibitor Fer-1 (1 μM). After 48 h incubation, cell viability was measured using CCK-8 assay.

### Proteome profiling of signaling pathways

To elucidate the signaling pathways, a proteome profiler for human phospho-kinases (ARY003C, R&D Systems, Minneapolis, MN, USA) was used. The levels of phosphorylation or expression were quantified using ImageJ software. The relative protein levels of phosphokinases were quantified using the reference protein spot.

### siRNA silencing

Cells were transfected with the designed siRNA oligonucleotides using Lipofectamine 3000 (Invitrogen, Carlsbad, CA, USA). The selected sequences of siRNAs were as follows: siCtrl:5′-TTCTCCGAACGTGTCACGT -3′, sip53: 5′-CACCATCCACTACAACTACAT-3′, and sip53-2: 5′GCACAGAGGAAGAGAATCT-3′.

### CRISPR/Cas9-based p53 knockout

CRISPR/Cas9 plasmid for p53 knockout was designed based on a pSpCas9(BB)-2A-Puro (PX462) plasmid with single-guide RNA (sgRNA). The CRISPR plasmid was electrotransfected into YT cells and selected using puromycin. The sequences of the sgRNA were used as follows: sgp53: 5′-GCAGTCACAGCACATGACGG-3′. 

### qRT-PCR

Total RNA was extracted using TRIzol reagent (Invitrogen, Carlsbad, CA, USA) and then reverse transcribed into cDNA using a FastQuant RT Kit (Tiangen, China). RT-PCR was performed using the TaqMan® Gene Expression Master Mix (Thermo Fisher Scientific, USA). The Glyceraldehyde-3-phosphate dehydrogenase (GAPDH) was used as the mRNA input control, and relative mRNA expression levels were computed using the 2^-Δ(CT)^ method. The primer sequences were shown below:

 p53-F:5′-TGGAGAATATTTCACCCTTCAGATC-3′;

p53-R:5′- TTTTTATGGCGGGAGGTAGACT-3′;

GAPDH-F:5′-CCTGCACCACCAACTGCTTA-3′;

GAPDH-R:5′-ATGGCATGGACTGTGGTCATG-3′.

### Western blot

YT cells were lysed with RIPA lysis buffer to prepare whole-cell extracts. Equal amounts of total protein (10 μg) were separated using sodium dodecyl sulfate–polyacrylamide gel electrophoresis, transferred onto polyvinylidene fluoride membrane (Pall Biotech., Westborough, MA, USA), and then probed with the appropriate primary and secondary antibodies. Immunodetection was performed using a ChemiDoc XRS + System (Bio-Rad, Hercules, CA, USA). The expression of the target protein was normalized to that of β-Actin (A5441, Sigma-Aldrich, St. Louis, MO, USA). Antibodies against PARP (9542, Cell Signaling Technology, Danvers, MA, USA), LC-3 (12,741, Cell Signaling Technology), phosphor-p53 (Ser46, 2521, Cell Signaling Technology), p53 (9282, Cell Signaling Technology), SLC7A11 (ab175186, Abcam Cambridge, MA, USA), and GPX4 (ab125066, Abcam) were used. Full-length blots of Western blot are presented in Supplementary materials WB Figures.

### Evaluation of synergism

After drug combination treatment and CCK-8 assay, the combination index (CI) of drug combinations under each experimental condition was calculated using the Compusyn software based on Chou-Talalay's median effect analysis [12]. CI > 1 indicates antagonistic, CI = 1 indicates additive, and CI < 1 indicates synergistic. The Synergy score was calculated based on ZIP reference model using SynergyFinder analysis (SynergyFinder) [13]. ZIP Synergy scores > 10 indicates synergistic, scores from -10 to 10 indicates additive, and scores < -10 indicates antagonistic.

### Statistical analysis

The results are presented as the mean ± standard deviation. Statistical analysis was conducted by the student-t test. A two-sided *P*-value of < 0.05 was considered to indicate a statistically significant difference. All analyses were performed using the SPSS software version 19.0 (IBM Corp., Armonk, NY, USA).

## Results

### Kayadiol inhibits the proliferation of NKTCL cells

**Fig. 1:**
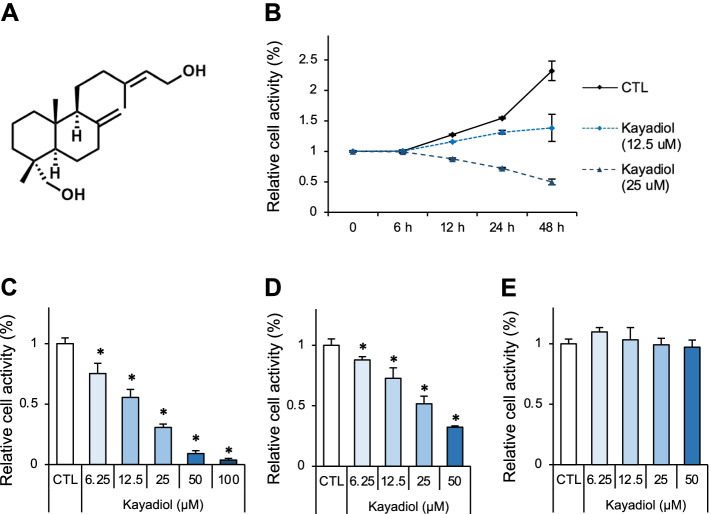
Proliferation of NKTCL cells incubated with different concentrations of Kayadiol, **A** Chemical structure of kayadio. **B** YT cells were treated with kayadiol for the indicated time points. **C** YT cells were treated with the designed concentration of kayadiol for 48 h. **D **and** E** PBLs from NKTCL patients (**D**) and healthy volunteers (**E**) were treated with the indicated concentration of kayadiol for 48 h. The cell viability was measured by the CCK-8 assay. The results are shown as the mean ± standard deviation, *p < 0.05

To observe the cytotoxicity and inhibitory effects of kayadiol in NKTCL cells, YT cells were stimulated with different concentrations of kayadiol at different time points. The chemical structure of kayadiol was shown in the Fig. 1A. Cell counting kit-8 assays showed that kayadiol suppressed YT cells’ growth in a dose- and time-dependent manner (Fig. 1B, C). Cell proliferation of PBLs from patients with NK/T cell lymphoma was also suppressed by kayadiol stimulation (Fig. 1D). However, kayadiol had no significant effect on cell growth of PBLs obtained from healthy donors (Fig. 1E). These results indicated that kayadiol had a killing effect on NKTCL cells.

### Kayadiol-induced cell death in NKTCL cells occurs through ferroptosis

To clarify the form of NKTCL cell death caused by kayadiol, we determined the expression of the apoptosis-related protein (cleaved-PARP) and autophagy-related protein (LC-3) after treatment with kayadiol. No significant effect on cleaved-PARP and LC-3 levels was observed by the stimulation of kayadiol, which indicated that other forms of cell death may have been accountable for the kayadiol-induced cell death.

**Fig. 2:**
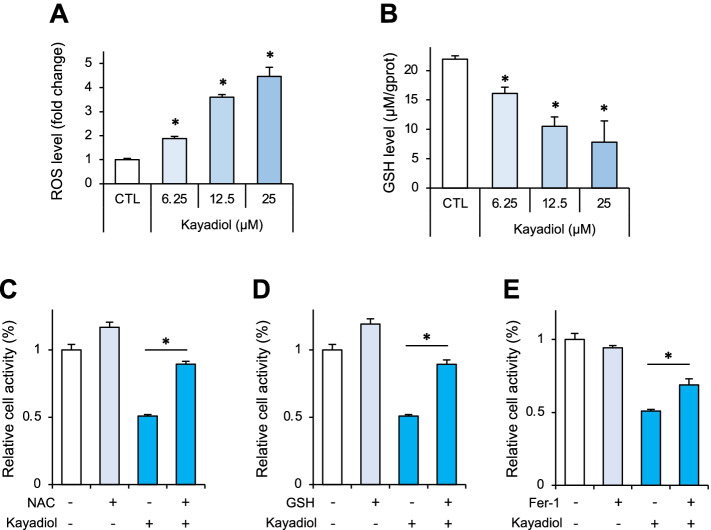
Kayadiol-induced cell death form in NKTCL cells, **A** Intracellular ROS levels in YT cells treated with kayadiol (12.5 μM) for 24 h, * *P* < 0.05 vs control group. **B** Intracellular GSH levels in YT cells treated with kayadiol (12.5 μM) for 24 h, * *P* < 0.05 vs control group. **C-E** YT cells were treated with kayadiol (12.5 μM) with or without ROS scavenger NAC (5 mM), reduced GSH (1 mM), or ferroptosis inhibitor Fer-1 (1 μM) for 48 h, and cell viability was measured. The results are shown as the mean ± standard deviation, * *P* < 0.05

We then evaluated whether ferroptosis, a newly discovered form of programmed cell death, was involved in kayadiol-induced cell death in NKTCL cells. It is well known that ROS accumulation and GSH depletion are typical iron-dependent ferroptosis events. We examined intracellular ROS and GSH levels following the treatment of kayadiol. As expected, the kayadiol stimulation elevated the ROS levels (Fig. 2A) and reduced the GSH levels (Fig. 2B). In addition, co-treatment with ROS scavenger NAC (Fig. 2C), reduced GSH (Fig. 2D), or ferroptosis inhibitor Fer-1 (Fig. 2E) rescued kayadiol-induced cell death.

### The p53 contributes to kayadiol-induced ferroptosis in NKTCL cells

**Fig. 3:**
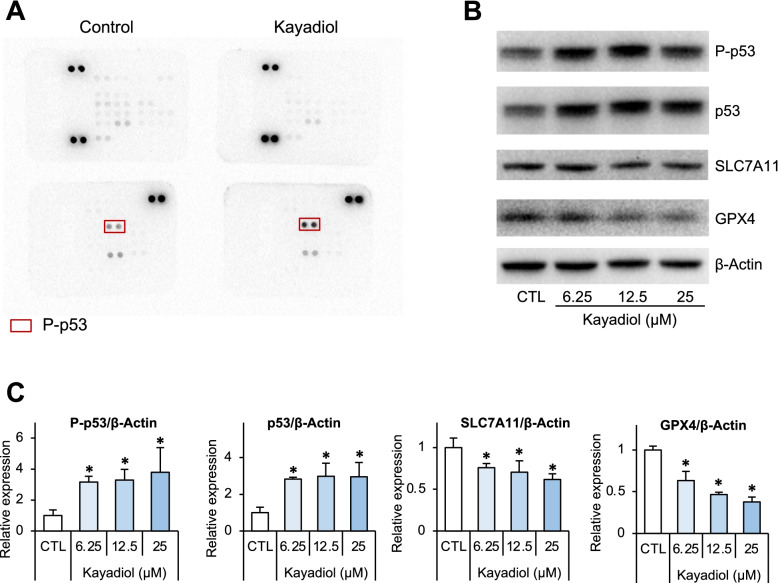
The target protein of kayadiol in NKTCL cells. YT cells were treated with kayadiol (12.5 μM) for 24 h. **A** Proteome profiling of signaling pathways showed p53 activation by kayadiol treatment in YT cells. **B** The expression of phosphorylated-p53, p53, SLC7A11, and GPX4 were examined by Western blotting, β-actin served as an endogenous control for normalization. **C** Quantification of the western blot signals was presented as means ± SD from 3 independent experiments, *P < 0.05 vs control group

To predict the targets of kayadiol in NKTCL cells, the antibody chip to explore the signaling pathway was used. Kayadiol promoted the phosphorylation of p53, without activating of AKT1, JAK-STAT, MAPK, and other signaling pathways (Fig. 3A). Western blot results confirmed that kayadiol significantly upregulated both phosphorylation of p53 and p53 protein expression (Fig. 3B, C). The role of p53 in ferroptosis was elucidated, and solute carrier family 7 member 11 (SLC7A11) was identified as a direct target gene suppressed by p53 [14]. GPX4 was also an import negative regulator of ferroptosis which could protect cells from lipid peroxidative damage [8, 9]. We found that kayadiol suppressed the expressions of these two negative regulators of ferroptosis, SLC7A11 and GPX4, in NKTCL cells (Fig. 3B, C).

**Fig. 4:**
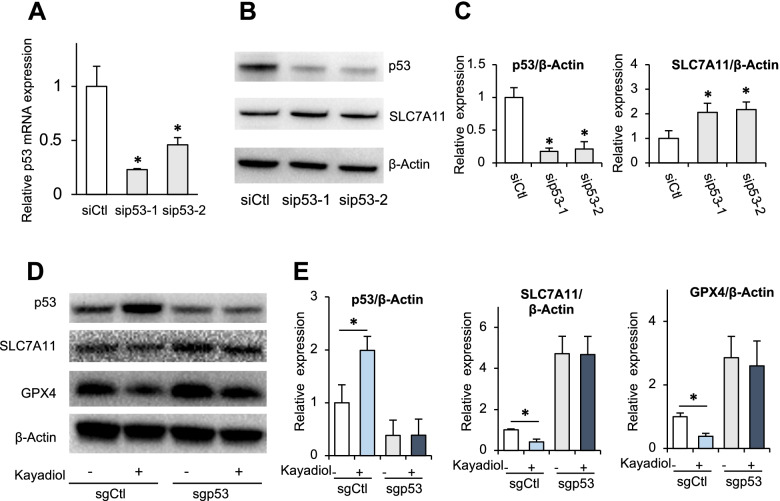
The regulatory role of p53 on kayadiol-induced ferroptosis in NKTCL cells, **A** Silencing of p53 by siRNAs decreased the expression of p53 at mRNA levels in YT cells. **B** Silencing of p53 by siRNAs decreased the protein expression of p53 in YT cells. **C** Expression levels of p53 and SLC7A11 were presented as means ± SD from 3 independent experiments, *P < 0.05 vi siCtl group. **D** Western blotting showed the effect of p53 knockout by CRISPR-Cas9 plasmid on kayadiol-induced ferroptosis in YT cells. **E** Quantification of the western blot signals was presented as means ± SD from 3 independent experiments, *P < 0.05

To confirm the causal role of p53 in kayadiol-induced ferroptosis, the expression of p53 in YT cells was silenced. A significant decreased mRNA and protein levels of p53 was observed in YT cells via transfecting sip53 (Fig. 4A, B and C). The protein levels of SLC7A11 were upregulated by p53 silencing (Fig. 4B and C). We then depleted p53 using CRISPR-Cas9 technology in YT cells and found a significant increase in the expression of SLC7A11 and GPX4. Kayadiol suppressed the expressions of SLC7A11 and GPX4 in YT cells, but on depleted p53 cells, no clear effect of kayadiol on SLC7A11 and GPX4 modulation are shown. These results indicated that kayadiol modulated p53, which in turn suppressed SLC7A11 and GPX4, in the absence p53, kayadiol is not expected to affect SLC7A11 and GPX4 levels (Fig. 4D, E). It confirmed that p53/SLC7A11/GPX4 signaling was a critical regulatory pathway of kayadiol-induced ferroptosis in NKTCL cells.

### Kayadiol exerts a synergistic effect with asparaginase and cisplatin in NKTCL cells

**Fig. 5:**
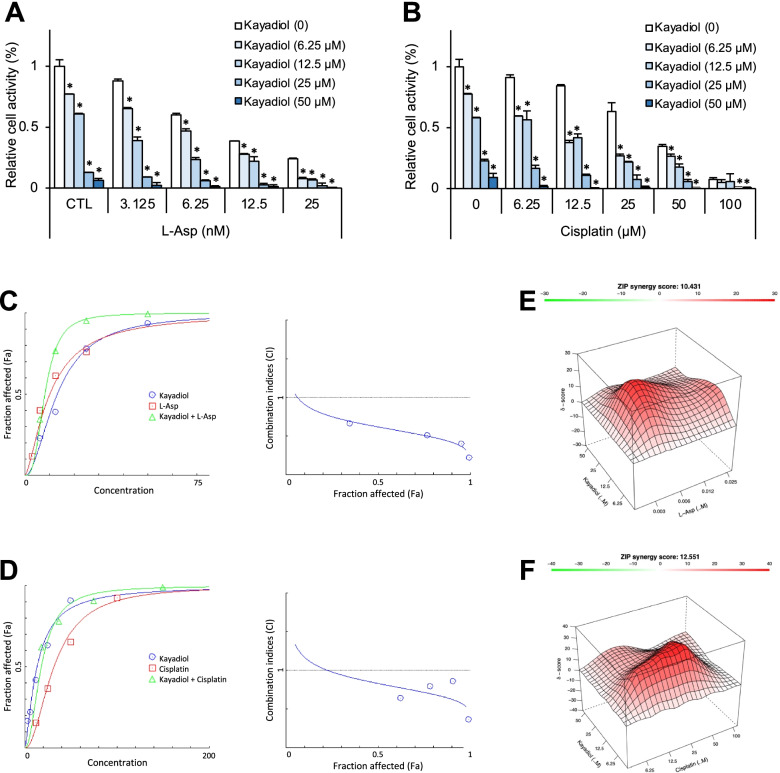
The combination efficacy of kayadiol with L-asparaginase or cisplatin in NKTCL cells, **A **and** B** YT cells were co-treated with kayadiol and L-Asp or ciaplatin for 48 h, and cell viability was assayed, * *P* < 0.05 vs kayadiol (0) group. **C **and** D** The CI plots of kayadiol and L-Asp or cisplatin were calculated using the Compusyn software based on Chou-Talalay method. **E **and** F** The synergy score of kayadiol with L-Asp or cisplatin was assayed by Synergyfinder software

It was reported that ferroptosis can reverse tumor cell resistance to cisplatin [15, 16]. We then evaluated the effect of kayadiol on the sensitivity of NKTCL cells to cisplatin and L-Asp, a main drug for lymphoma chemotherapy regimen. The CCK8 results showed that kayadiol significantly increased the sensitivity of YT cells to L-Asp (Fig. 5A) and cisplatin (Fig. 5B). To detect the synergistic efficacy, we used the Chou-Talalay method for analysis, and the combination index (CI) plots of two drugs were calculated using the Compusyn software. The results indicated the synergistic effect of kayadiol with L-Asp or cisplatin (CI < 1, Fig. 5C, D). Furthermore, we used the Synergyfinder analysis to detect the synergy effect again, which further confirmed the synergistic effect of kayadiol with L-Asp or cisplatin (Synergy scores > 10, Fig. 5E, F). These results suggested that kayadiol had a synergistic effect with L-Asp and cisplatin, which may play an important role in reversing chemotherapy resistance in NKTCL cells.

## Discussion

The value of natural compounds in the treatment of human diseases has been well documented. The use of natural products and their metabolites in enhancing physiological functions and treating pathological conditions doubled the human lifespan during the twentieth century [17]. Terpenoids, the largest class of natural products, exert strong anticancer activities [4, 18]. For example, paclitaxel, a diterpenoid isolated from *Taxus wallichiana*, has been widely used in the clinical treatment of breast cancer, ovarian cancer, and non-small cell lung cancer [19, 20]. Although the anticancer effects of terpenoids have been studied for years, there are still many kinds of terpenoids that have not been comprehensively studied. In this study, we screened some rarely reported natural terpenoids and found that kayadiol, a diterpenoid extracted from *Torreya nucifera*, exerted a significant killing effect on NKTCL cells without killing healthy lymphocytes.

It is well known that T cells and NK cells are key players of the immune system to kill cancer cells. The most prestigious tumor immunotherapy, chimeric antigen receptor-engineered T cells (CAR-T), was also developed based on the tumor-killing function of healthy T cells. NK/T cell lymphoma originated from cancerous NK cells or T cells, and the loss of normal NK/T cell functions make NK/T cell lymphoma more aggressive and difficult to treat than other malignant tumors. So far, there is no therapy format proved best in the treatment of NK/T cell lymphoma [2]. Exploring drugs that can target and kill tumor cells without affecting normal NK/T cells is useful in managing this malignant disease. Kayadiol exactly exerted such a function.

In this research, we reported that p53-mediated ferroptosis occurred in kayadiol-induced cell death in NKTCL cells. It is well known that p53 exhibits tumor suppression through conventional functions such as cell cycle arrest, apoptosis, senescence, and autophagy. Accumulating evidence demonstrated the important role of p53 in ferroptosis, a newly identified form of regulated cell death [14, 21]. SLC7A11, the first identified direct target gene of p53 [22], is an important member of the cystine/glutamate exchange transporter system (xCT system) and promotes the exchange of intracellular glutamate for extracellular cystine [23]. Under the catalysis of γ-glutamylcysteine synthetase and glutathione synthetase, cystine synthesizes GSH. GPX4 uses GSH to reduce lipid hydroperoxides to non-toxic lipid alcohols, thereby suppressing ferroptosis [24, 25]. In our study, kayadiol promoted p53 expression, which repressed SLC7A11 and affected the synthesis of GSH, thus decreasing the activity of GPX4 and resulting in ferroptosis cell death. Of note, kayadiol could induce cell death in both YT cells and PBLs from NKTCL patients, but not in PBLs from healthy donors, implying that lymphoma cells are more sensitive to kayadiol than lymphocytes from healthy donor. The molecular mechanism underlying this phenomenon is unclear, one possibility is that kayadiol expert anti-cancer effect on NKTCL cells through p53, and *TP53* mutation was reported in 20%–60% of NKTCL cases, which might make the cancerous NK or T cells more sensitive to kayadiol than healthy lymphocytes [26–28]. Although further research is required to test this hypothesis, our findings seem to support the clinical application potential of kayadiol in NKTCL.

Chemoresistance is one of the main treatment problems in NK/T cell lymphoma, especially in advanced disease [29]. Ferroptosis is reported to be of great significance in reversing resistance to chemotherapies in cancers. *Sato *et al. showed that pretreatment with erastin, an inducer of ferroptosis, strongly elevated the sensitivity to cisplatin in tumor cells [15]. *Roh *et al*.* reported that inhibition of Keap1-Nrf2, a key pathway of the cellular oxidative stress response, induces ferroptosis and reverses the resistance of cisplatin-resistant in head and neck tumor cells [16]. *Fu* et al. developed an Fe(VI)-based nanocomposite system, which can convert H_2_O_2_ into active hydroxyl radicals in tumor cells, induce ferroptosis and improve tumor sensitivity to tondoxorubicin in solid tumors [30]. In our research, kayadiol significantly increased the sensitivity of YT cells to L-Asp and cisplatin, which significantly improved its clinical application value.

In summary, our study reported for the first time that kayadiol had the potential to induce ferroptosis in NKTCL cells without affecting health lymphocytes, which made it a potential compound for NKTCL treatment. Additional in vivo and large-scale clinical investigations should be conducted in the future to substantiate these research findings.

## Supplementary Information

**Additional file 1.**

**Additional file 2: Supplement Figure 1.** The effect of kayadiol on cleaved-PARP and LC-3 levels in NKTCL cells. (A) The expression of apoptosis-related protein (cleaved-PARP) and autophagy-related protein (LC-3) were examined by Western blotting, β-actin served as an endogenous control for normalization. (B) Quantification of the western blot signals was presented as means ± SD from 3 independent experiments.

## Data Availability

All data generated or analyzed during this study are included in this published article.

## Abbreviations

NKTCL: Extranodal natural killer /T-cell lymphoma
L-Asp: L-asparaginase
OS: Overall survival
ROS: Reactive oxygen species
GPX4: Glutathione peroxidase
GSH: Glutathione
NAC: N-Acetylcysteine
PBL: Peripheral blood lymphocytes
CCK-8: Cell counting kit-8
siRNA: Small interfering RNA
sgRNA: Single-guide RNA
qRT-PCR: Quantitative Real-Time polymerase chain reaction
SLC7A11: Solute carrier family 7 member 11
CI: Combination index
CAR-T: Chimeric antigen receptor-engineered T cells
xCT system: Cystine/glutamate exchange transporter system

## References

[1] Wang L, Wang JW (2020). Extranodal natural-killer T-cell lymphoma: experience from China. Lancet Haematol..

[2] Wang H, Fu BB, Gale RP, Liang Y (2021). NK-/T-cell lymphomas. Leukemia..

[3] Rodrigues T, Reker D, Schneider P, Schneider G (2016). Counting on natural products for drug design. Nat Chem..

[4] El-Baba C, Baassiri A, Kiriako G, Dia B, Fadlallah S, Moodad S, Darwiche N (2021). Terpenoids' anti-cancer effects: focus on autophagy. Apoptosis..

[5] Chen SP, Dong M, Kita K, Shi QW, Cong B, Guo WZ, Sugaya S, Sugita K, Suzuki N (2010). Anti-proliferative and apoptosis-inducible activity of labdane and abietane diterpenoids from the pulp of Torreya nucifera in HeLa cells. Mol Med Rep..

[6] Dixon SJ, Lemberg KM, Lamprecht MR, Skouta R, Zaitsev EM, Gleason CE, Patel DN, Bauer AJ, Cantley AM, Yang WS (2012). Ferroptosis: an iron-dependent form of nonapoptotic cell death. Cell..

[7] Chen X, Li J, Kang R, Klionsky DJ (2021). Ferroptosis: machinery and regulation. Autophagy.

[8] Yang WS, SriRamaratnam R, Welsch ME, Shimada K, Skouta R, Viswanathan VS, Cheah JH, Clemons PA, Shamji AF, Clish CB (2014). Regulation of ferroptotic cancer cell death by GPX4. Cell..

[9] Ursini F, Maiorino M (2020). Lipid peroxidation and ferroptosis: The role of GSH and GPx4. Free Radic Biol Med..

[10] Zou Y, Palte MJ, Deik AA, Li H, Eaton JK, Wang W, Tseng YY, Deasy R, Kost-Alimova M, Dancik V (2019). A GPX4-dependent cancer cell state underlies the clear-cell morphology and confers sensitivity to ferroptosis. Nat Commun..

[11] Jiang X, Stockwell BR, Conrad M (2021). Ferroptosis: mechanisms, biology and role in disease. Nat Rev Mol Cell Biol..

[12] Chou TC (2010). Drug combination studies and their synergy quantification using the Chou-Talalay method. Cancer Res..

[13] Ianevski A, Giri AK, Aittokallio T (2020). SynergyFinder 2.0: visual analytics of multi-drug combination synergies. Nucleic Acids Res.

[14] Liu Y, Gu W (2022). p53 in ferroptosis regulation: the new weapon for the old guardian. Cell Death Differ.

[15] Sato M, Kusumi R, Hamashima S, Kobayashi S, Sasaki S, Komiyama Y, Izumikawa T, Conrad M, Bannai S, Sato H (2018). The ferroptosis inducer erastin irreversibly inhibits system xc- and synergizes with cisplatin to increase cisplatin's cytotoxicity in cancer cells. Sci Rep..

[16] Roh JL, Kim EH, Jang H, Shin D. Nrf2 inhibition reverses the resistance of cisplatin-resistant head and neck cancer cells to artesunate-induced ferroptosis. Redox Biol. 2017;11:254–62. 10.1016/j.redox.2016.12.010. 10.1016/j.redox.2016.12.010 PMC5198738 28012440

[17] Demain AL (2006). From natural products discovery to commercialization: a success story. J Ind Microbiol Biotechnol..

[18] Gershenzon J, Dudareva N (2007). The function of terpene natural products in the natural world. Nat Chem Biol..

[19] Yang YH, Mao JW, Tan XL (2020). Research progress on the source, production, and anti-cancer mechanisms of paclitaxel. Chin J Nat Med..

[20] Abu Samaan TM, Samec M, Liskova A, Kubatka P, Busselberg D (2019). Paclitaxel's mechanistic and clinical effects on breast cancer. Biomolecules..

[21] Lei G, Zhang Y, Hong T, Zhang X, Liu X, Mao C, Yan Y, Koppula P, Cheng W, Sood AK (2021). Ferroptosis as a mechanism to mediate p53 function in tumor radiosensitivity. Oncogene..

[22] Jiang L, Kon N, Li T, Wang SJ, Su T, Hibshoosh H, Baer R, Gu W (2015). Ferroptosis as a p53-mediated activity during tumour suppression. Nature..

[23] Koppula P, Zhuang L, Gan B (2021). Cystine transporter SLC7A11/xCT in cancer: ferroptosis, nutrient dependency, and cancer therapy. Protein Cell..

[24] FriedmannAngeli JP, Schneider M, Proneth B, Tyurina YY, Tyurin VA, Hammond VJ, Herbach N, Aichler M, Walch A, Eggenhofer E (2014). Inactivation of the ferroptosis regulator Gpx4 triggers acute renal failure in mice. Nat Cell Biol..

[25] Sui X, Zhang R, Liu S, Duan T, Zhai L, Zhang M, Han X, Xiang Y, Huang X, Lin H (2018). RSL3 drives Ferroptosis through GPX4 inactivation and ROS production in colorectal cancer. Front Pharmacol..

[26] Aozasa K, Takakuwa T, Hongyo T, Yang WI (2008). Nasal NK/T-cell lymphoma: epidemiology and pathogenesis. Int J Hematol..

[27] Lee S, Park H, Kang S, Kim S, Hwang J, Lee S, Kwak S, Park K, Yoo H, Kim WS (2015). Genetic alterations of JAK/STAT cascade and histone modification in extranodal NK/T-cell lymphoma nasal type. Oncotarget.

[28] Jiang L, Gu Z, Yan Z, Zhao X, Xie Y, Zhang Z, Pan C, Hu Y, Cai C, Dong Y (2015). Exome sequencing identifies somatic mutations of DDX3X in natural killer/T-cell lymphoma. Nat Genet..

[29] Bi XW, Jiang WQ, Zhang WW, Huang JJ, Xia Y, Wang Y, Sun P, Li ZM (2015). Treatment outcome of patients with advanced stage natural killer/T-cell lymphoma: elucidating the effects of asparaginase and postchemotherapeutic radiotherapy. Ann Hematol..

[30] Fu J, Li T, Yang Y, Jiang L, Wang W, Fu L, Zhu Y, Hao Y (2021). Activatable nanomedicine for overcoming hypoxia-induced resistance to chemotherapy and inhibiting tumor growth by inducing collaborative apoptosis and ferroptosis in solid tumors. Biomaterials..

