# Supplementary material for: Kayadiol exerted anticancer effects through p53-mediated ferroptosis in NKTCL cells
Source: BMC Cancer. 2022 Jul 2;22:724. doi: 10.1186/s12885-022-09825-5 (PMC9250166; doi:10.1186/s12885-022-09825-5)
Supplement: Supplementary file 1 — Additional file 1. [file 12885_2022_9825_MOESM1_ESM.pdf]

Full-length blots of Figure 3B

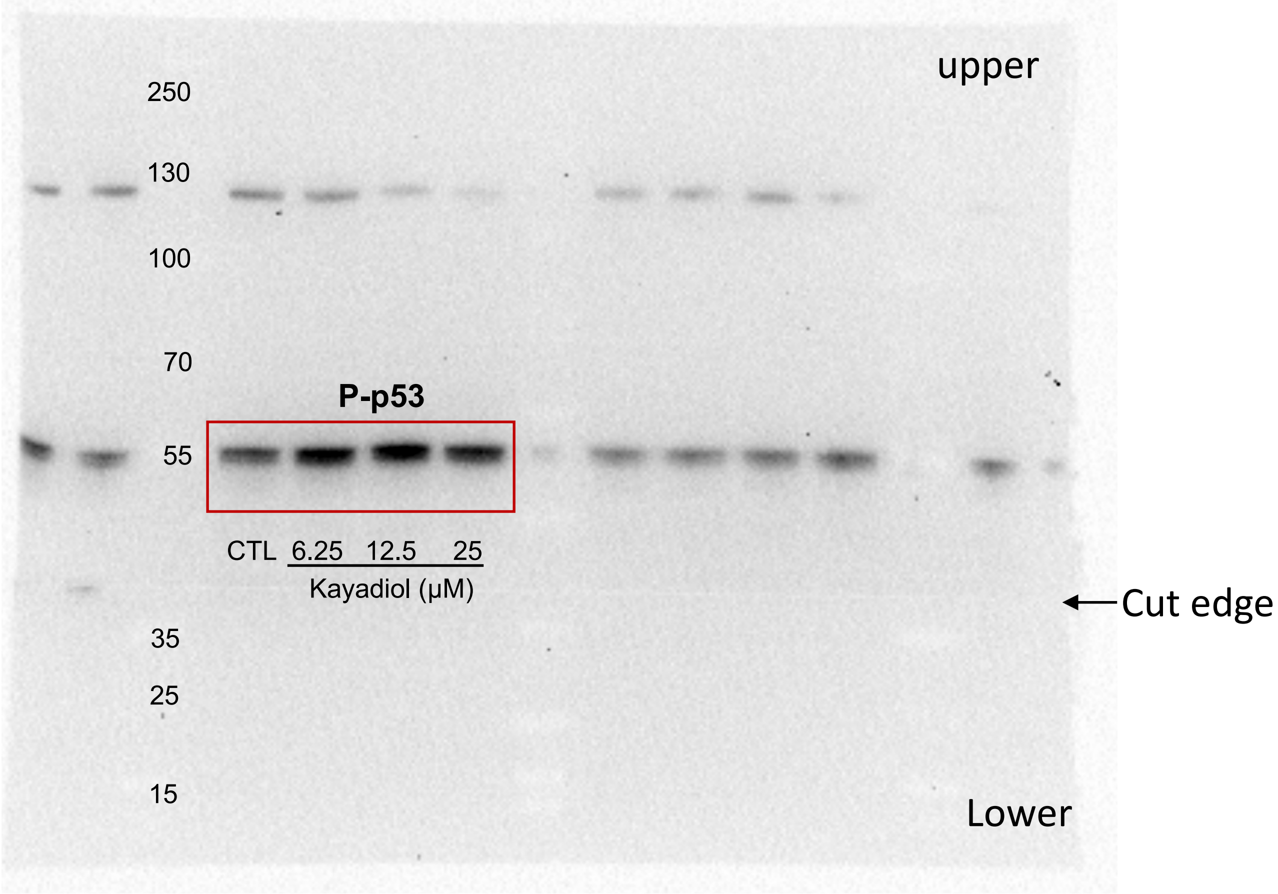

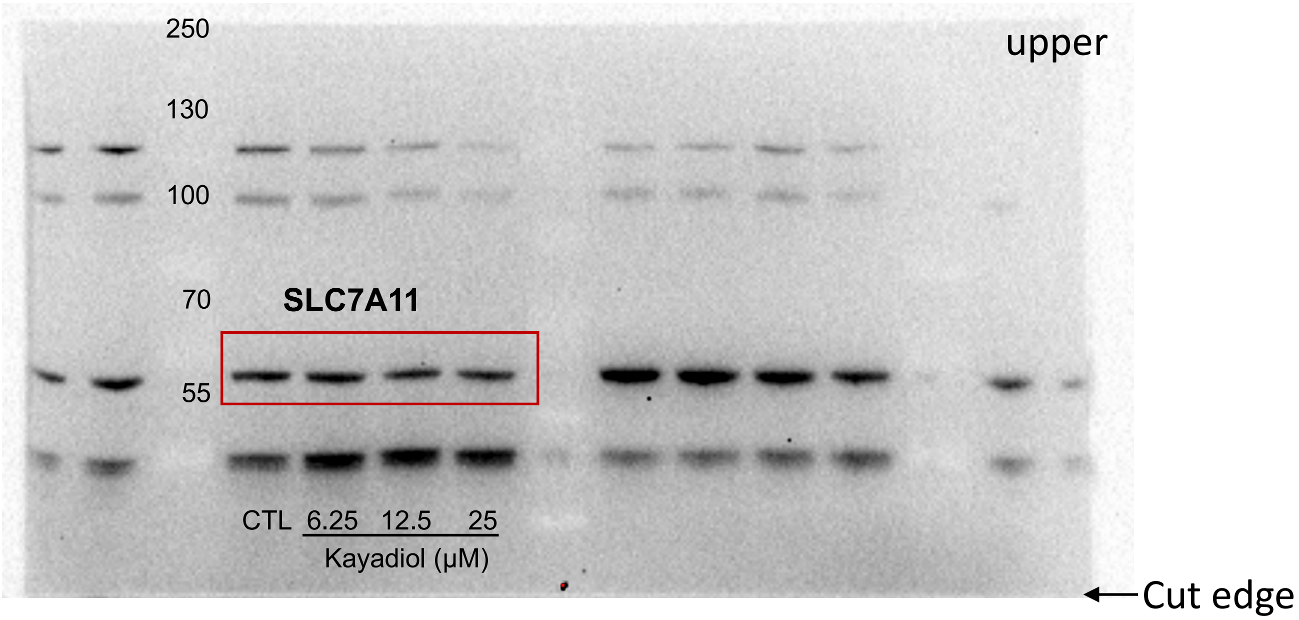

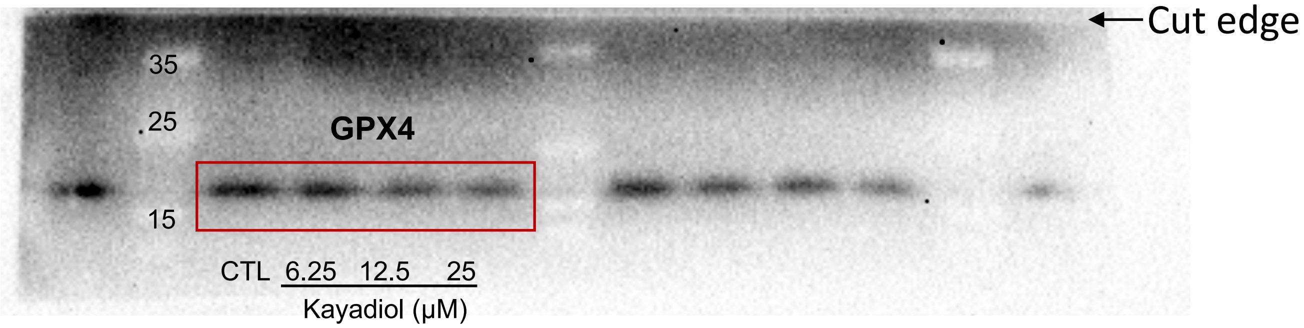

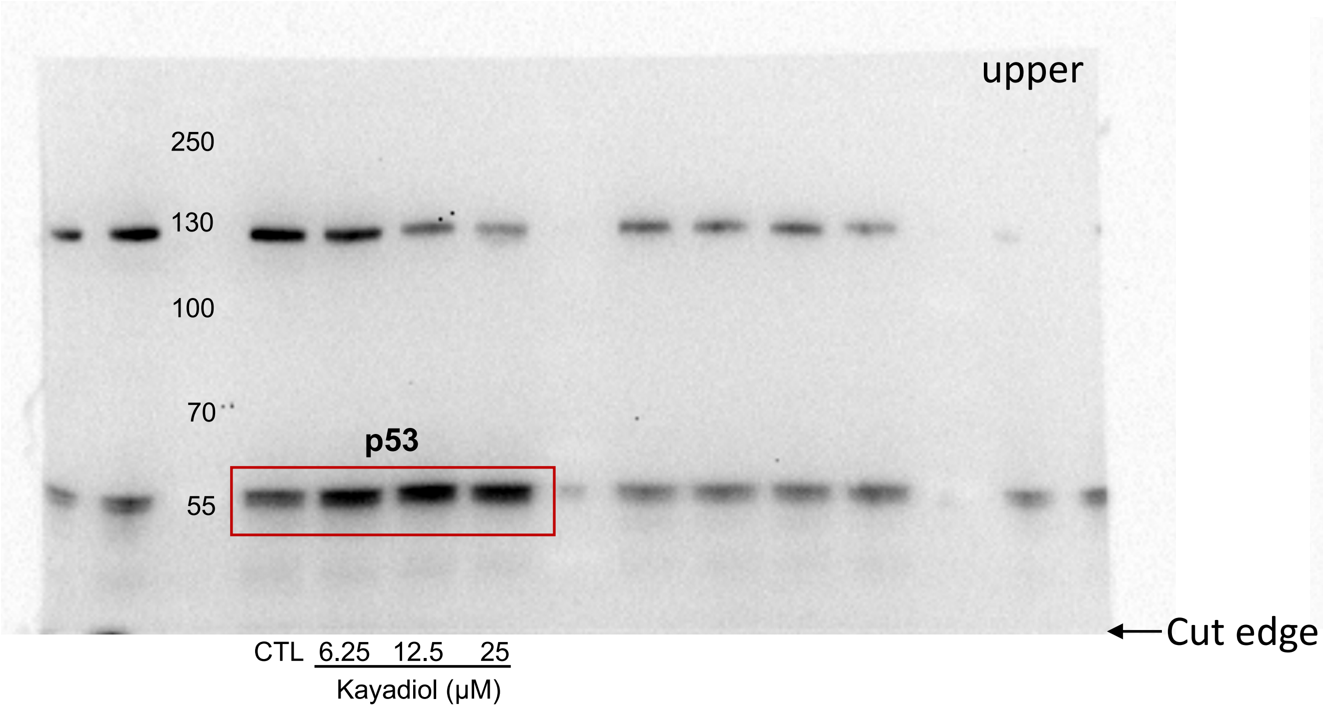

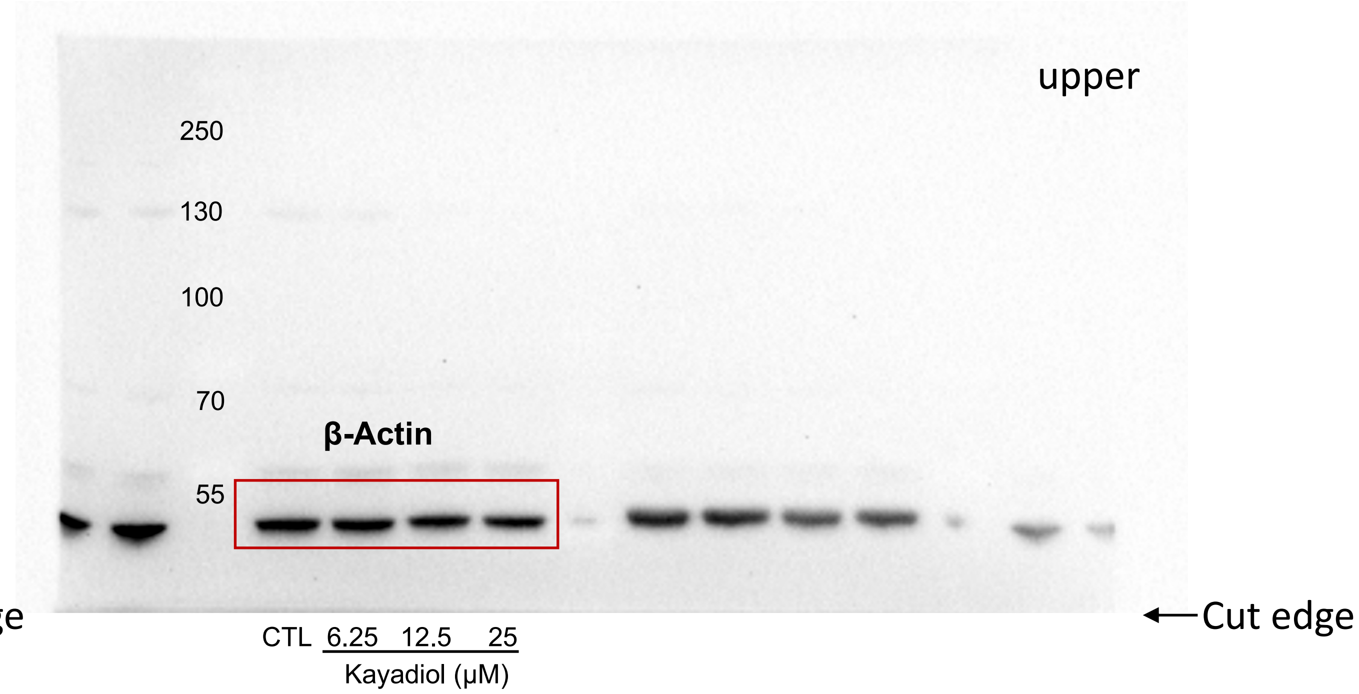

The full-length blots of figure 3B: Western blot for p-p53, p53, SLC7A11, GPX4, and β-Actin from protein lysates of YT cells .

Full-length blots of Figure 4B

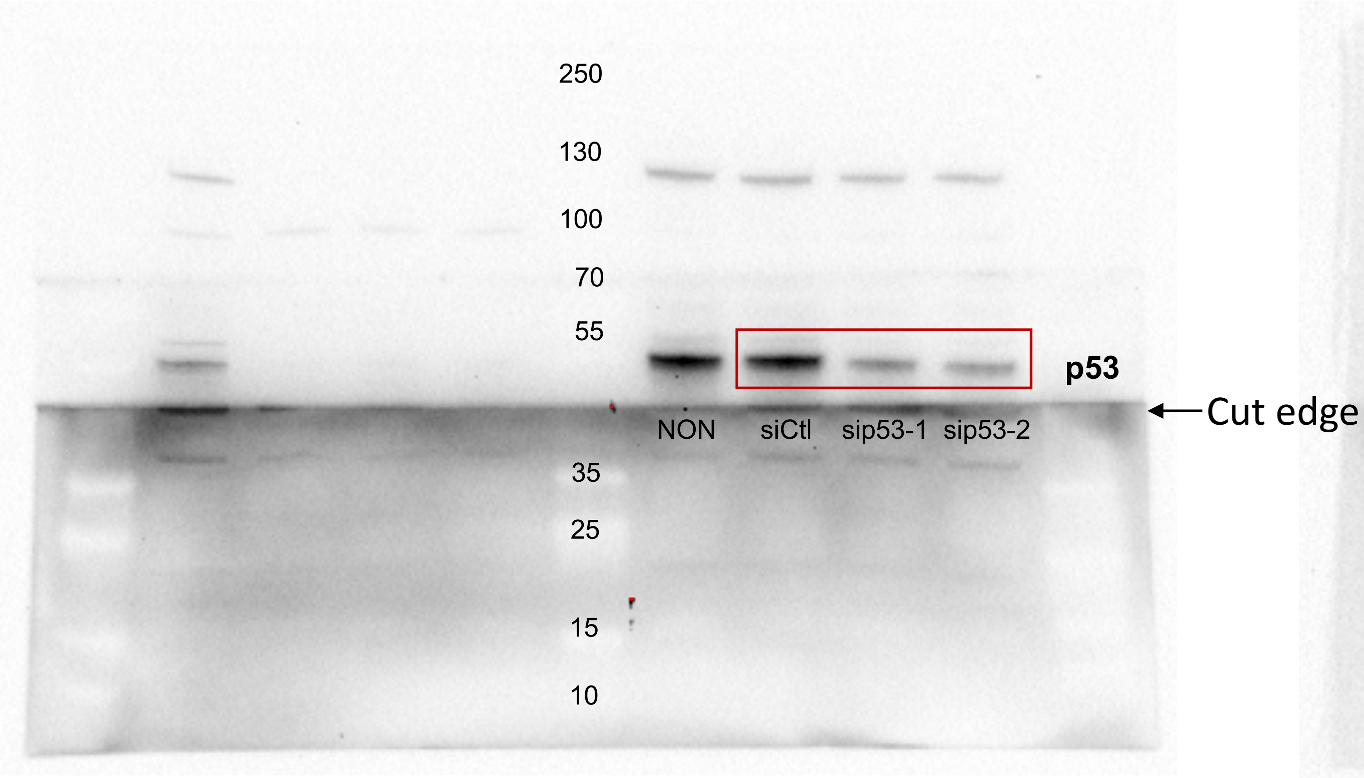

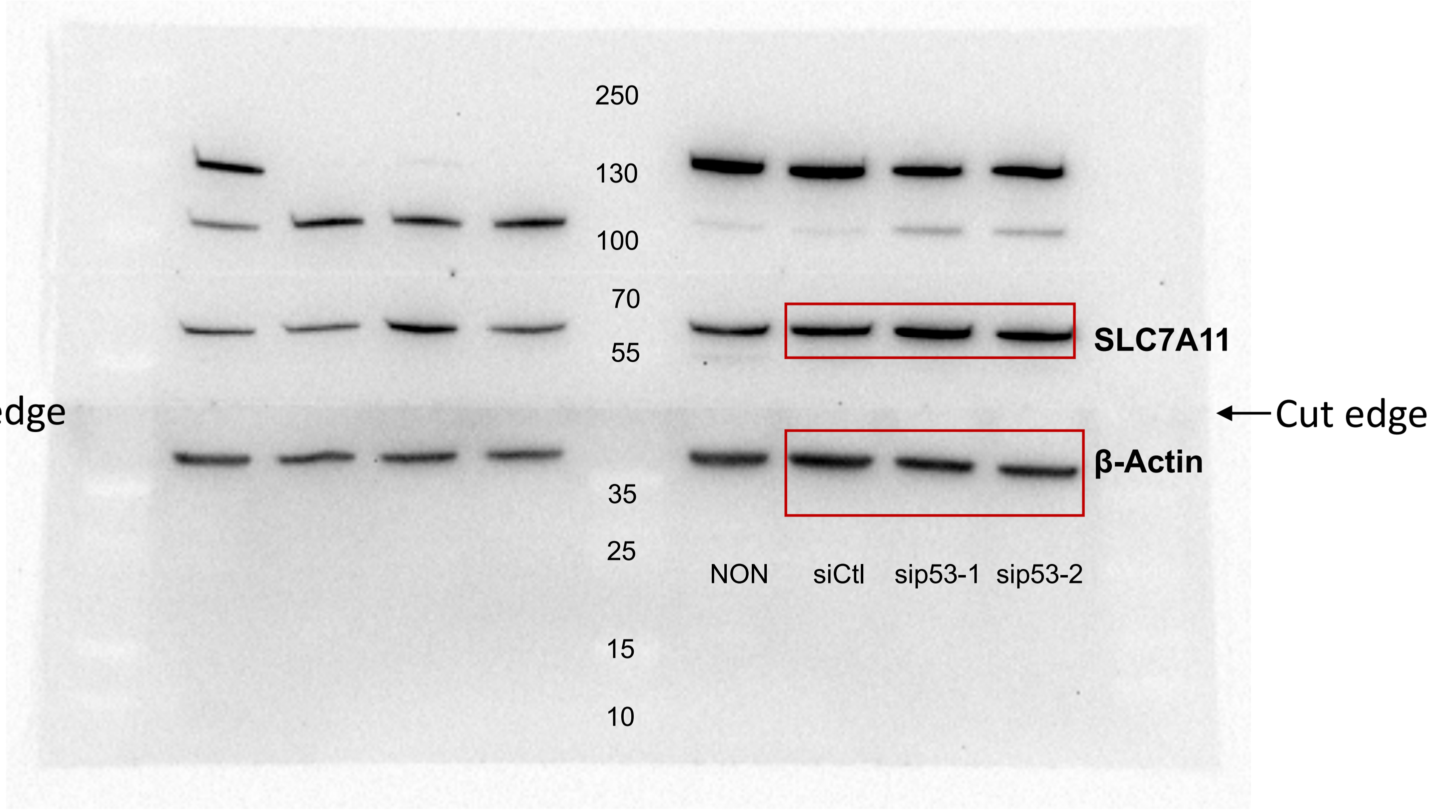

The full-length blots of figure 4B: Western blot for p53, SLC7A11, and β-Actin from protein lysates of YT cells .

Full-length blots of Figure 4D

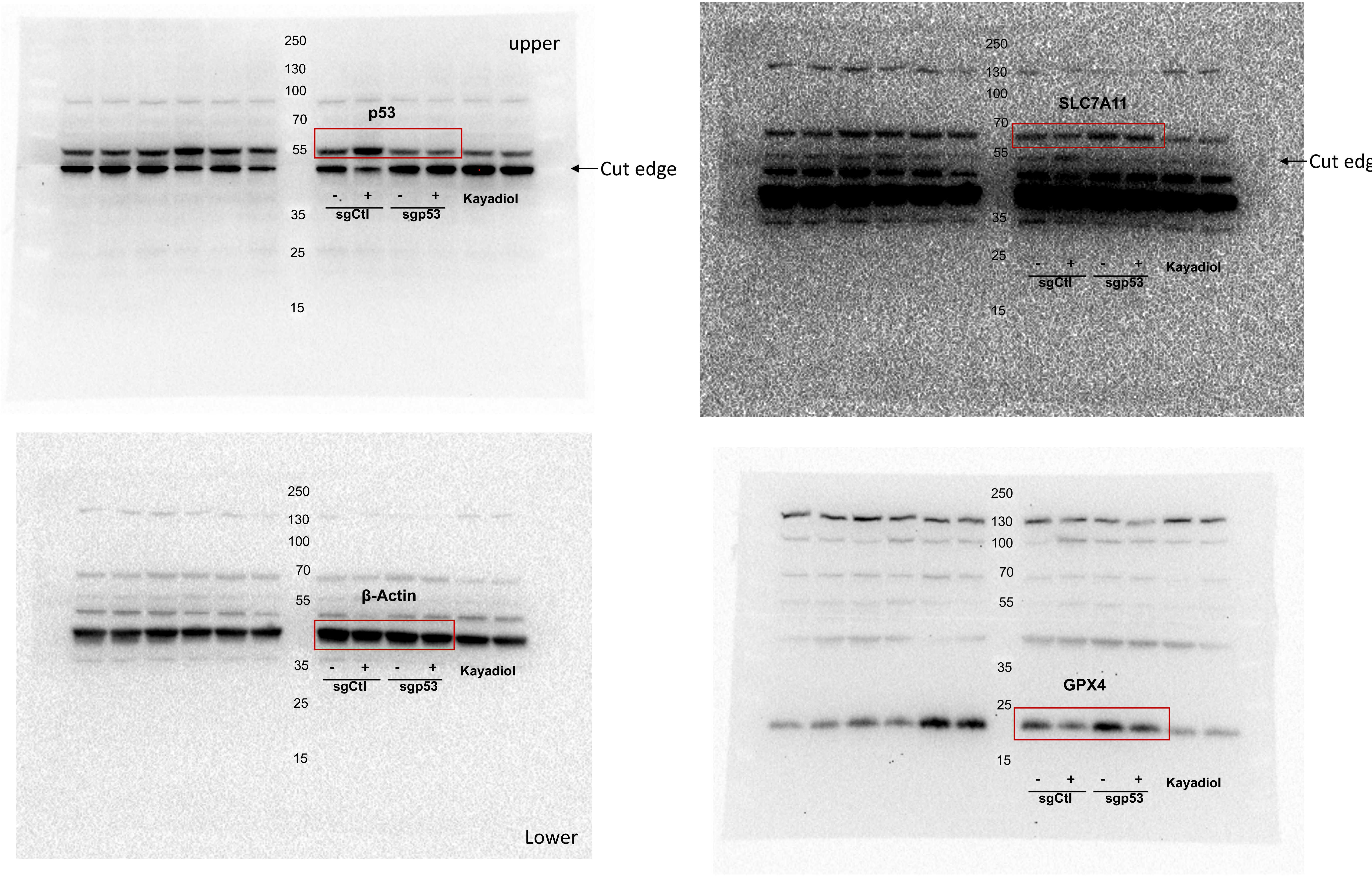

The full-length blots of figure 4B: Western blot for p53, SLC7A11, GPX4, and β-Actin from protein lysates of YT cells .

Full-length blots of Sup Figure. 1

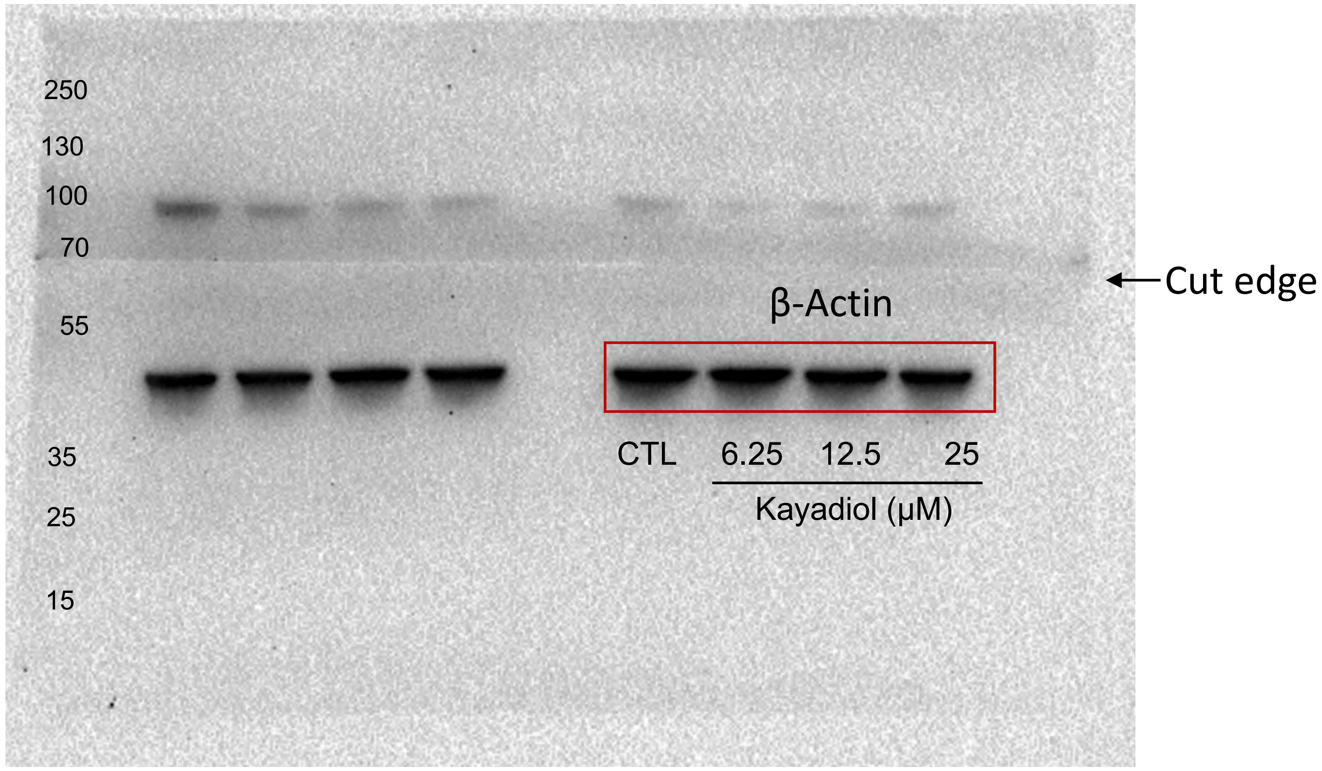

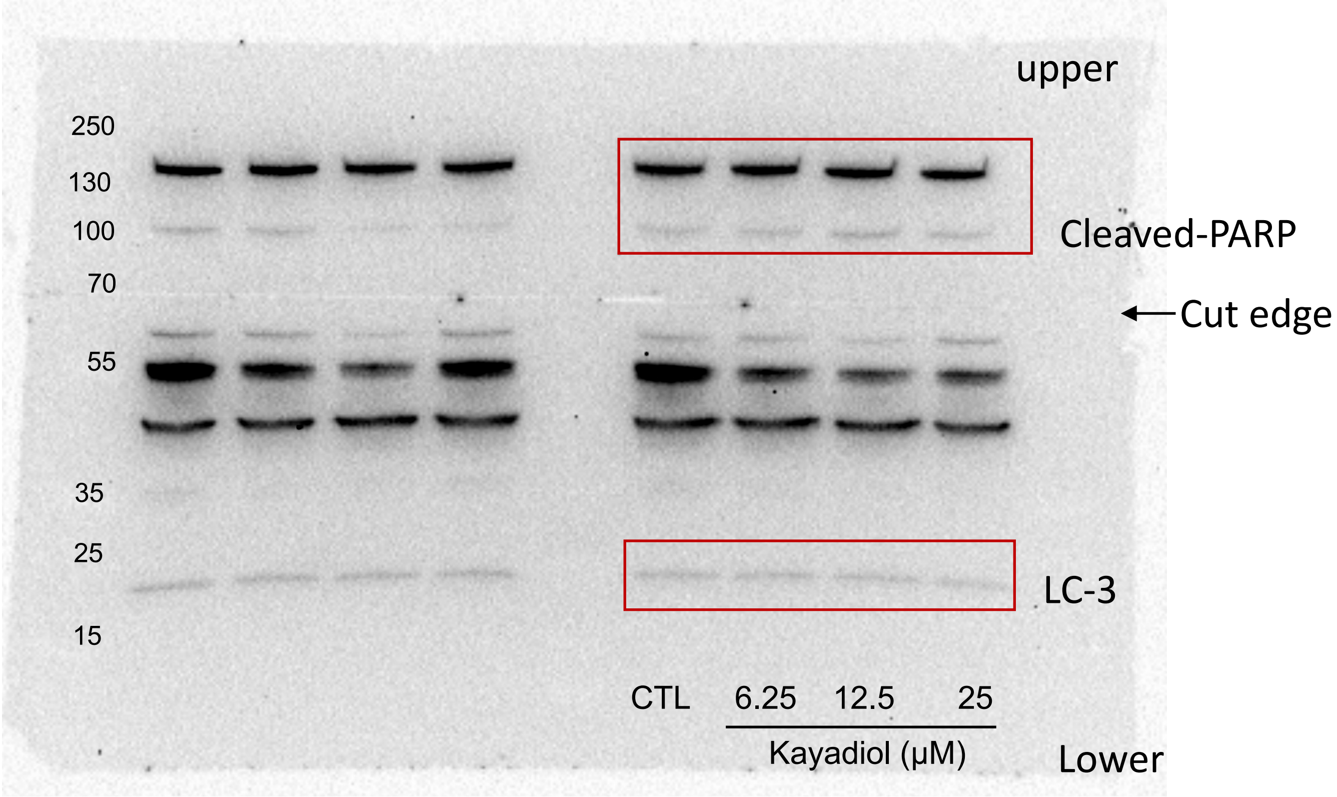

The full-length blots of figure 4B: Western blot for PARP, LC-3, and β-Actin from protein lysates of YT cells .
