# Supplementary figures and images for: Kayadiol exerted anticancer effects through p53-mediated ferroptosis in NKTCL cells
Source: BMC Cancer. 2022 Jul 2;22:724. doi: 10.1186/s12885-022-09825-5 (PMC9250166; doi:10.1186/s12885-022-09825-5)

Sup Fig. 1

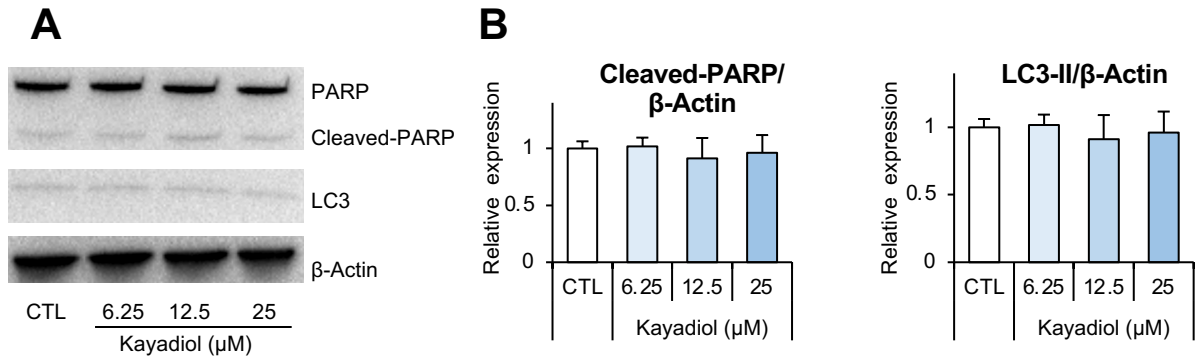

Supplement: Supplementary file 2 — Additional file 2: Supplement Figure 1. The effect of kayadiol on cleaved-PARP and LC-3 levels in NKTCL cells. (A) The expression of apoptosis-related protein (cleaved-PARP) and autophagy-related protein (LC-3) were examined by Western blotting, β-actin served as an endogenous control for normalization. (B) Quantification of the western blot signals was presented as means ± SD from 3 independent experiments. [file 12885_2022_9825_MOESM2_ESM.pdf]
